# Supplementary material for: Identifying depression subtypes and investigating their consistency and transitions in a 1-year cohort analysis
Source: PLoS One. 2025 Jan 14;20(1):e0314604. doi: 10.1371/journal.pone.0314604 (PMC11731715; doi:10.1371/journal.pone.0314604)
Supplement: S1 Table — The table represents the demographic and clinical sample characteristics at baseline. (PDF) [file pone.0314604.s001.pdf]

S.1 Table

Demographic and Baseline Sample Characteristics

Variable	Total Sample (N=619)
Gender: *n* (%)	
Male	151 (24.4)
Female	468 (75.6)
Study Site: *n (%)*	
London	347 (56.1)
Barcelona	154 (24.9)
Amsterdam	118 (19.1)
Age: mean (S.D.)	46.3 (15.2)
Aggregated Ethnicity: *n (%)*	
White British/Dutch	368 (79.1)
White Other	35 (7.5)
Black ethnic group	14 (3.0)
Asian ethnic group	16 (3.4)
Mixed ethnic background	14 (3.0)
Other	18 (3.9)
Current depression (IDS-SR)	
IDS-SR total, *M(SD)*	31.3 (14.5)
None (0–13), *N(%)*	61 (9.9)
Mild (14–25), *N(%)*	157 (25.4)
Moderate (26–38), *N(%)*	206 (33.3)
Severe (39–48), *N(%)*	104 (16.8)
Very severe (49–84), *N(%)*	79 (12.8)
Not reported *N(%)*	12 (19.4)
Suicidal ideation, N(%)	
Yes	110 (17.7)
Taking antidepressants, N(%)	
Yes	407 (65.8)
Selective serotonin reuptake inhibitor (SSRI)	265 (42.8)
Serotonin–norepinephrine reuptake inhibitor (SNRI)	130 (21.0)
Mirtazapine	42 (6.8)
Antipsychotics	55 (8.9)

Note. Ethnicity data not collected at Spanish site (N = 155). Ethnicity data aggregated according to recommendations provided here: https://www.ethnicity-facts-figures.service.gov.uk/style-guide/writing-about-ethnicity. Antidepressant groups are not exclusive, participants might be taking multiple. IDS-SR: Inventory of Depressive Symptomatology – Self Report. M(SD): Mean (Standard Deviation).
