# Supplementary material for: Identifying depression subtypes and investigating their consistency and transitions in a 1-year cohort analysis
Source: PLoS One. 2025 Jan 14;20(1):e0314604. doi: 10.1371/journal.pone.0314604 (PMC11731715; doi:10.1371/journal.pone.0314604)
Supplement: S2 Table — The results presented in S2 Table, outline the local dependence violations seen in the 3 and 4-class solutions for baseline, 6-months and 12-months. Baseline results are discussed in the results section. For 6 months the original 3 and 4 class solutions (i.e., no modelled dependence) presented with 2 and 1 pairs of problematic local dependence between the appetite and weight variables, respectively. In both solutions modelling the dependence removed the concerns. Meanwhile, for the 12-month model violations were only observed in the three-class solution, while the 4-class solution presented no violations. (PDF) [file pone.0314604.s002.pdf]

S2.1 Table

Local independence violations with a Person test statistic >15 for the 3 and 4 class LCA Models at Baseline, 6-months, and 12-months.

		Baseline N=619		6-Months N=542		12-Months N=432	
k	Variable Pairs	Base model	Partial Dependence Model	Base model	Partial Dependence Model	Base model	Partial Dependence Model
3 Class	Appetite Increase & Weight Increase	37.27	0	92.82	0	31.91	unknown
	Appetite Decrease & Weight Decrease	0	17.04	22.03	0	23.00	unknown
4 Class	Appetite Increase & Weight Increase	20.71	0	0	0	0	NA
	Appetite Decrease & Weight Decrease	0	0	22.34	0	0	NA
	Weight Decrease & Weight Increase	0	0	0	0	0	NA

Notes. The violations defined in the base models (i.e., no modelled dependence) are then accounted for in the partial dependence model. Unknown = model did not terminate normally; NA =no partial dependence model was run.
