# Supplementary material for: Identifying depression subtypes and investigating their consistency and transitions in a 1-year cohort analysis
Source: PLoS One. 2025 Jan 14;20(1):e0314604. doi: 10.1371/journal.pone.0314604 (PMC11731715; doi:10.1371/journal.pone.0314604)
Supplement: S3 Table — (PDF) [file pone.0314604.s003.pdf]

S3.1 Table

Probabilities of Endorsing Depressive Symptoms Derived from 4-Class Baseline Latent Class Analysis (N=619)

	**Class 1**	**Class 2**	**Class 3**	**Class 4**
Class description	Severe with Appetite Decrease	Severe with Appetite Increase	Moderate	Low
Prevalence, %	12.3	12.4	36.0	39.3
Mood	0.89	0.94	0.51	0.03
Insomnia	0.63	0.54	0.45	0.34
Hypersomnia	0.27	0.31	0.13	0.04
Appetite Decrease	0.52	0.04	0.10	0.02
Appetite Increase	0.02	0.36	0.10	0.07
Weight Decrease	0.41	0.00	0.13	0.10
Weight Increase	0.00	0.37	0.10	0.07
Lack of Concentration	0.91	0.88	0.44	0.01
Guilt/Worthlessness	0.83	0.71	0.45	0.13
Suicidal	0.39	0.38	0.19	0.04
Lack of Interest	0.78	0.73	0.23	0.03
Energy Loss	0.86	1.00	0.53	0.06
Psychomotor Retardation	0.89	0.49	0.19	0.07
Psychomotor Agitation	0.63	0.22	0.15	0.10
