# Supplementary material for: Identifying depression subtypes and investigating their consistency and transitions in a 1-year cohort analysis
Source: PLoS One. 2025 Jan 14;20(1):e0314604. doi: 10.1371/journal.pone.0314604 (PMC11731715; doi:10.1371/journal.pone.0314604)
Supplement: S5 Table — The results in S5 Table show that the 4-Class solution presented a good middle ground between the 3 and 5-Class solutions across the timepoints. Specifically, at 6-months, 4-class solution presented the best model fit for BIC and featured only a slightly higher aBIC than the 5-class solution. Furthermore, entropy was higher for the 4-class than the 5-class or 6-class solution, as such this was chosen. Similarly at 12-month follow-up, 4-class solution was chosen as it has the best fitting aBIC value, furthermore it has considerably higher entropy than the 6-class solution. The results for baseline are outlined in the results section. (PDF) [file pone.0314604.s005.pdf]

S4.1 Table

Model Comparisons in Cross-Sectional Latent Class Analysis at baseline, 6-Month and 12-Month follow-up

k	AIC	BIC	Adj. BIC	Entropy
Baseline (N=619)				
2	8141	8269	8177	**0.835**
3	8058	**8253**	8113	0.801
4	8007	8268	8081	0.746
5	7978	8305	**8070**	0.737
6	**7968**	8363	8080	0.772
6 months (N=542)				
2	7188	7313	7221	**0.834**
3	7084	7273	7134	0.833
4	7015	**7268**	7081	0.820
5	6979	7297	**7062**	0.792
6	**6965**	7348	7065	0.796
12 months (N=432)				
2	5573	5692	5600	**0.850**
3	5501	**5682**	5542	0.759
4	5466	5708	**5520**	0.761
5	5461	5765	5530	0.722
6	**5458**	5823	5540	0.748

Note. k= the number of latent classes in the model; AIC = Akaike information criterion; BIC= Bayesian information criterion; Adj. BIC= sample-adjusted BIC.
