# Supplementary material for: Identifying depression subtypes and investigating their consistency and transitions in a 1-year cohort analysis
Source: PLoS One. 2025 Jan 14;20(1):e0314604. doi: 10.1371/journal.pone.0314604 (PMC11731715; doi:10.1371/journal.pone.0314604)
Supplement: S6 Table — Here we can see that overall, the derived model shares a similar interpretation to the baseline latent class model and the 6-, 12-month models. It identifies four classes (1) severe with appetite decrease, (2) severe with appetite increase, (3) moderate severity, (4) low severity. Measurement invariance was assessed by comparing model fit in a nested model comparison. Here the restricted model (item response probabilities held equal over time) was preferred over the freely varying model. Specifically, the AIC, BIC and aBIC were lower for the restricted model (ΔAIC = -211.76, ΔBIC = -1203.65, ΔaBIC = -492.493). This assumption was also formally tested using a nested loglikelihood ratio test (G22 –G21 = 118.12, df = 224, p>0.05) further suggesting measurement invariance. (PDF) [file pone.0314604.s006.pdf]

S5.1 Table

Probabilities of Endorsing Depressive Symptoms Derived from One-Step Measurement Invariant Latent Transition Analysis Model: Baseline, 3-, 6-, 9-, 12- month (N=619)

	**Class 1**	**Class 2**	**Class 3**	**Class 4**
Class description	Severe with Appetite Decrease	Severe with Appetite Increase	Moderate	Low
Mood	0.93	0.80	0.51	0.05
Insomnia	0.66	0.45	0.43	0.25
Hypersomnia	0.27	0.32	0.15	0.04
Appetite Decrease	0.39	0.00	0.09	0.01
Appetite Increase	0.02	0.75	0.09	0.08
Weight Decrease	0.28	0.02	0.11	0.08
Weight Increase	0.06	0.63	0.10	0.11
Lack of Concentration	0.92	0.84	0.45	0.07
Guilt/Worthlessness	0.82	0.82	0.47	0.08
Suicidal	0.39	0.32	0.19	0.04
Lack of Interest	0.79	0.67	0.28	0.02
Energy Loss	0.92	0.96	0.55	0.08
Psychomotor Retardation	0.73	0.49	0.19	0.04
Psychomotor Agitation	0.53	0.24	0.21	0.11
