# Supplementary material for: Identifying depression subtypes and investigating their consistency and transitions in a 1-year cohort analysis
Source: PLoS One. 2025 Jan 14;20(1):e0314604. doi: 10.1371/journal.pone.0314604 (PMC11731715; doi:10.1371/journal.pone.0314604)
Supplement: S7 Table — Interpretation of these probabilities suggests that membership in all 4 classes was stable over time and participants were most likely to stay within their same class over time, with probabilities ranging from 94% chance for remaining in class 4 from 9 to 12 months, and 68% chance for remaining in class 3 from baseline to 3 months. Notable between class changes over-time occur for individuals moving from class 2 to class 3. Here those in class 2 at report a 27% chance of moving to class 3 from 3 to 6 months and from 6 to 9 months. (PDF) [file pone.0314604.s007.pdf]

S5.2 Table

Transition probabilities and latent class sizes for one-step LTA model for baseline, 3-, 6- , 9-, & 12-months (N=619)

	**Class 1** Severe w/ App. Decrease	**Class 2** Severe w/ App. Increase	**Class 3** Moderate	**Class 4** Low
Class sizes for each latent class per timepoint				
Baseline	6.7%	17.0%	35.8%	40.5%
3-months	8.6%	17.5%	34.7%	39.2%
6-months	6.7%	18.9%	35.8%	38.6%
9-months	6.8%	17.1%	34.8%	41.3%
12-months	7.7%	16.1%	33.1%	43.1%
Transition probabilities from baseline classes (rows) to 3-months (columns)				
Class 1: Severe w/ App. Decrease	**0.79**	0.06	0.15	0.00
Class 2: Severe w/ App. Increase	0.15	**0.69**	0.09	0.07
Class 3: Moderate	0.09	0.08	**0.68**	0.15
Class 4: Low	0.00	0.00	0.18	**0.82**
Transition probabilities from 3-months classes (rows) to 6-months (columns)				
Class 1: Severe w/ App. Decrease	**0.85**	0.06	0.08	0.01
Class 2: Severe w/ App. Increase	0.08	**0.60**	0.27	0.06
Class 3: Moderate	0.09	0.01	**0.81**	0.09
Class 4: Low	0.01	0.00	0.10	**0.89**
Transition probabilities from 6-months classes (rows) to 9-months (columns)				
Class 1: Severe w/ App. Decrease	**0.86**	0.00	0.14	0.00
Class 2: Severe w/ App. Increase	0.00	**0.73**	0.27	0.00
Class 3: Moderate	0.02	0.05	**0.78**	0.16
Class 4: Low	0.01	0.01	0.06	**0.92**
Transition probabilities from 9-months classes (rows) to 12-months (columns)				
Class 1: Severe w/ App. Decrease	**0.82**	0.03	0.10	0.05
Class 2: Severe w/ App. Increase	0.00	**0.83**	0.00	0.17
Class 3: Moderate	0.05	0.05	**0.84**	0.07
Class 4: Low	0.01	0.00	0.05	**0.94**

Note. Probabilities of staying in the same class are marked in bold.
