# Supplementary material for: Identifying depression subtypes and investigating their consistency and transitions in a 1-year cohort analysis
Source: PLoS One. 2025 Jan 14;20(1):e0314604. doi: 10.1371/journal.pone.0314604 (PMC11731715; doi:10.1371/journal.pone.0314604)
Supplement: S8 Table — (PDF) [file pone.0314604.s008.pdf]

S6.1 Table

Transition probabilities and class sizes for three-step LTA model for baseline, 6-months, 12-months.

	**Class 1** Severe w/ App. Decrease	**Class 2** Severe w/ App. Increase	**Class 3** Moderate	**Class 4** Low
Class sizes for each latent class per timepoint				
Baseline	12%	9%	36%	43%
6-months	14%	9 %	38%	38%
12-months	13%	12%	30%	46%
Transition probabilities from baseline classes (rows) to 6-months (columns)				
Class 1: Severe w/ App. Decrease	**0.77**	0.10	0.13	0.00
Class 2: Severe w/ App. Increase	0.14	**0.61**	0.17	0.08
Class 3: Moderate	0.10	0.07	**0.71**	0.12
Class 4: Low	0.00	0.00	0.23	**0.78**
Transition probabilities from 6-months classes (rows) to 12-months (columns)				
Class 1: Severe w/ App. Decrease	**0.77**	0.10	0.13	0.00
Class 2: Severe w/ App. Increase	0.00	**0.70**	0.08	0.22
Class 3: Moderate	0.04	0.08	**0.62**	0.25
Class 4: Low	0.00	0.02	0.10	**0.88**

Note. Probabilities of staying in the same class are marked in bold.
