# Supplementary material for: Identifying depression subtypes and investigating their consistency and transitions in a 1-year cohort analysis
Source: PLoS One. 2025 Jan 14;20(1):e0314604. doi: 10.1371/journal.pone.0314604 (PMC11731715; doi:10.1371/journal.pone.0314604)
Supplement: S9 Table — (PDF) [file pone.0314604.s009.pdf]

S7.1 Table

Descriptive crosstabulation of class membership at baseline in relation to participant characteristics

Predictor Present	**Class 1 N= 53** Severe w/ Appetite Decrease	**Class 2 N=30** Severe w/ Appetite Increase	**Class 3 N=161** Moderate	**Class 4 N=188** Low
Gender *n*(%)				
Male	13 (11.8)	4 (3.6)	43 (39.1)	50 (45.5)
Female	40 (12.4)	26 (8.1)	118 (36.6)	138 (42.9)
Age (years/10) mean (S.D.)	4.4 (1.4)	4.5 (1.5)	4.5 (1.5)	5.0 (1.5)
Family history of MDD *n*(%)				
No	20 (17.1)	9 (7.7)	50 (42.7)	38 (32.5)
Yes	33 (10.5)	21 (6.7)	111 (35.2)	150 (47.6)
Physical health comorbidity *n*(%)				
No	21 (8.3)	16 (6.3)	91 (36.0)	125 (49.4)
Yes	32 (17.9)	14 (7.8)	70 (39.1)	63 (35.2)
Mental health comorbidity *n*(%)				
No	9 (6.1)	6 (4.1)	57 (38.5)	76 (51.4)
Yes	44 (15.5)	24 (8.5)	104 (36.6)	112 (39.4)
Cardiometabolic comorbidity *n*(%)				
No	44 (11.8)	27 (7.2)	142 (38.1)	160 (42.9)
Yes	9 (15.3)	3 (5.1)	19 (32.2)	28 (47.5)
Lifetime traumatic events *n*(%)				
No	38 (12.7)	19 (6.4)	115 (38.5)	127 (42.5)
Yes	15 (11.5)	11 (8.4)	44 (33.6)	61 (46.6)
NA	0 (0.0)	0 (0.0)	2 (100)	0 (0.0)
SSRI *n*(%)				
No	31 (12.5)	17 (6.9)	94 (37.9)	106 (42.7)
Yes	22 (12.0)	13 (7.1)	67 (36.4)	82 (44.6)
SNRI *n*(%)				
No	34 (9.9)	20 (5.8)	125 (36.2)	166 (48.1)
Yes	19 (21.8)	10 (11.5)	36 (41.4)	22 (25.3)
Antipsychotics *n*(%)				
No	40 (10.4)	27 (7.0)	142 (36.9)	176 (45.7)
Yes	13 (27.7)	3 (6.4)	19 (40.4)	12 (25.5)
Mirtazapine *n*(%)				
No	47 (11.7)	27 (6.7)	146 (36.4)	181 (45.1)
Yes	6 (19.4)	3 (9.7)	15 (48.4)	7 (22.6)

Note. N=432. Ns shown represent class sizes in the model without any covariates included in the model. Antidepressant & comorbidity groups are not exclusive. Age: in decades to create more interpretable coefficients. Selective serotonin reuptake inhibitor: SSRI; Serotonin–norepinephrine reuptake inhibitor: SNRI. Lifetime traumatic events, scoring >6 was considered high number of traumatic events.
