# Supplementary material for: Identifying depression subtypes and investigating their consistency and transitions in a 1-year cohort analysis
Source: PLoS One. 2025 Jan 14;20(1):e0314604. doi: 10.1371/journal.pone.0314604 (PMC11731715; doi:10.1371/journal.pone.0314604)

S2.1 Fig

Item-response Probabilities for Endorsing Depressive Symptoms at Baseline in 4 Class Partial Dependence Model and 4 Class Base Model (no modelled dependence)

4 Class Partial Dependence Model

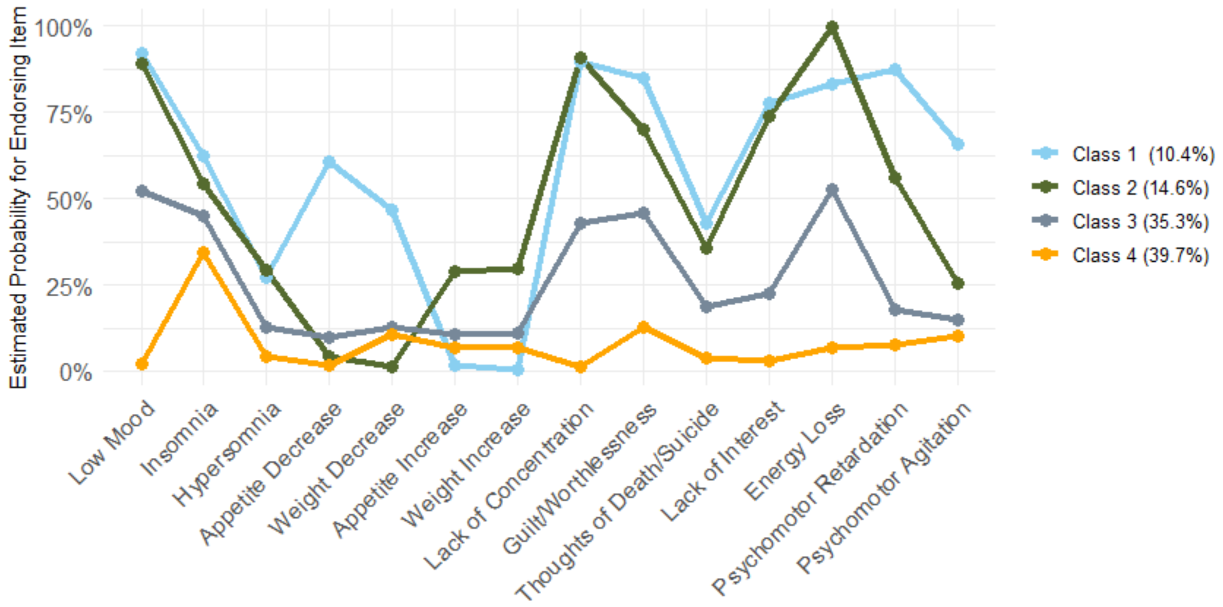

4 Class Base Model (no modelled dependence)

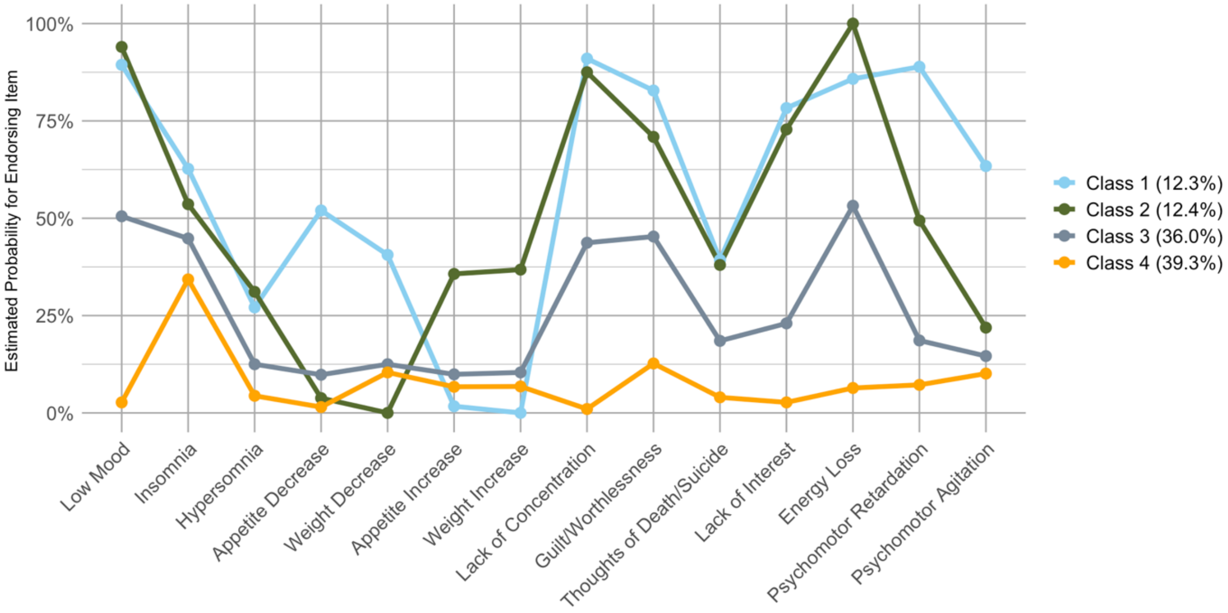

Supplement: S1 Fig — (PDF) [file pone.0314604.s010.pdf]
