# Supplementary material for: Identifying depression subtypes and investigating their consistency and transitions in a 1-year cohort analysis
Source: PLoS One. 2025 Jan 14;20(1):e0314604. doi: 10.1371/journal.pone.0314604 (PMC11731715; doi:10.1371/journal.pone.0314604)

S4.1 Figure

Item-response Probabilities for Endorsing Depressive Symptoms at baseline, 6-Month and 12-Month follow-up

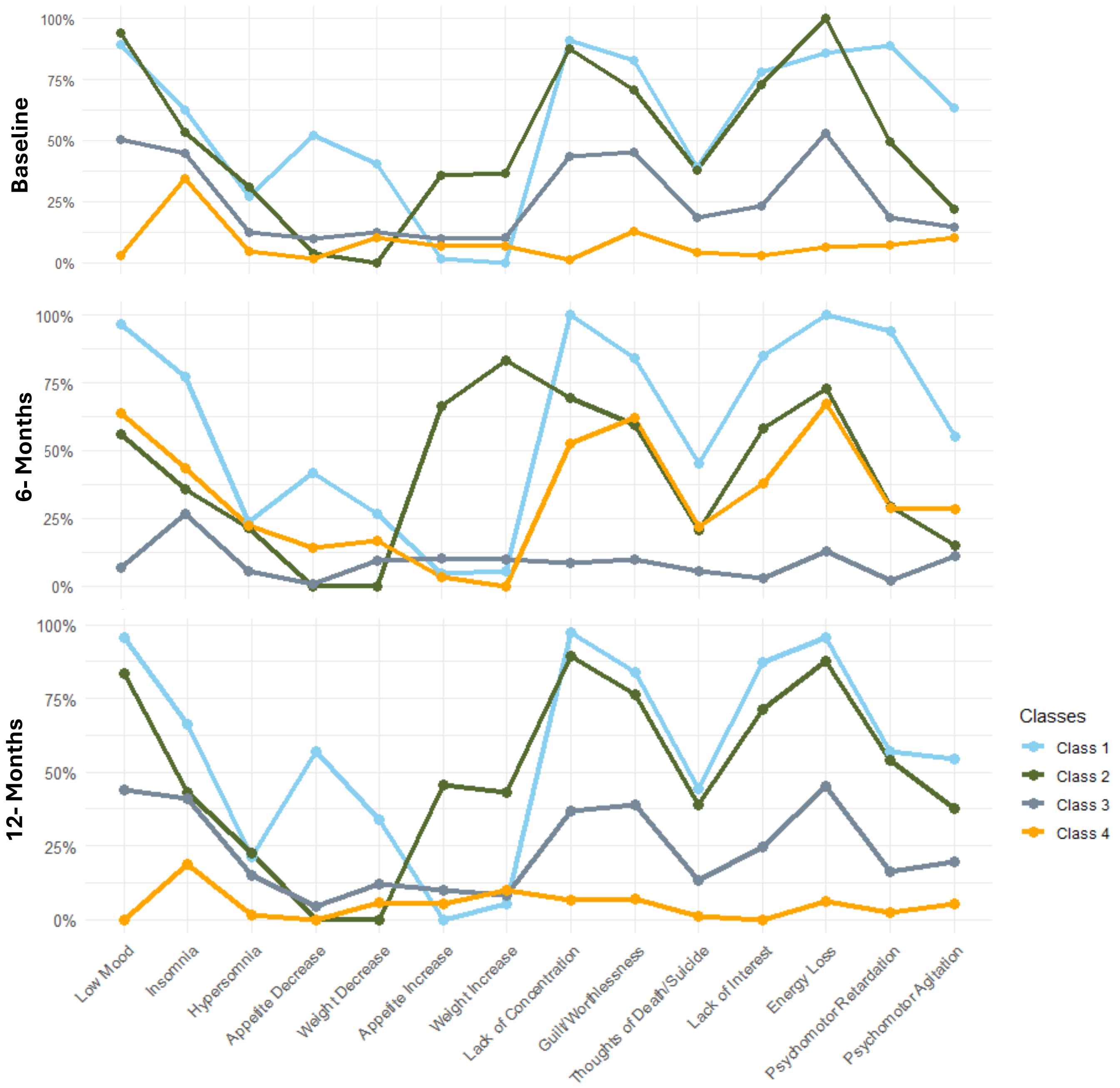

Supplement: S2 Fig — The results presented in S2 Fig, show that across each of the independently run latent class analyses, the item-response probabilities for endorsing a symptom are comparable in the 4-class solutions over time. (PDF) [file pone.0314604.s011.pdf]
