# Supplementary material for: Identifying depression subtypes and investigating their consistency and transitions in a 1-year cohort analysis
Source: PLoS One. 2025 Jan 14;20(1):e0314604. doi: 10.1371/journal.pone.0314604 (PMC11731715; doi:10.1371/journal.pone.0314604)
Supplement: Attachment — Submitted filename: Reviewer_comments_v2.docx [file pone.0314604.s012.docx]

Dear Esteemed Editor and Reviewers,

Thank you for the detailed and constructive feedback on our manuscript. We are pleased to provide an amended version addressing all comments raised by the reviewers. Please find our point-by-point responses below.

**Reviewer #1
Strengths**
1. Well written introduction that clearly outlines the contribution of this work.
2. Thank you for sharing your code, that is an important step to ensure reproducibility.
3. Good use of a relatively real-world dataset.
4. Great coaching of results with respect to other literature and past work, e.g. the Van Loo et al statement.
5. Very well written and easy to read.

**Response:** Thank you for your positive comments.

Minor Comments
**1. Please mention your specific multiple comparison correction in the text, I found it only in a table caption.**

**Response:** Thank you for your comment. We have clarified the relevant statement in the Methods section to specify the type of comparison completed.

*“Corrections for multiple comparisons were made using the Benjamin-Hochberg ^38^ method in R ^39^.”*

**2. There is a missing closing paren on lines 38 or 39.**

**Response:** Thank you for your comment. We have corrected the missing parenthesis on line 70.

**3. Is there a legend for Figure 1, or otherwise somewhere where we can understand which group is which?**

**Response:** Apologies, this seemed to have been missed. We have added a legend to the figure, labelled Figure 2 in the revised manuscript.

**Reviewer #2:** 
**1. Introduction: Line 29, it’s not mentioned which version of DSM you are referring to.**

**Response:** Thank you for your comments and apologies for the oversight. We have revised this line to clarify:

*“Recent attempts have aimed at identifying new phenotypic classes or verifying classes defined by the DSM-5.”*

**2. Method: I would prefer to see a figure here, where the participant flow is shown, drop-out etc**.

**Response:** Thank you for your suggestion, we have now added a flowchart of participant follow-up (figure 1).

**3. Results: Line 201, figure 1 is not presented in the text. It makes it hard to understand the differences between the different groups. I think it also would be wise to go more in depth in the different symptoms of depression that you are investigating.**

**Response:** Thank you for your comment. We apologise for omitting Figure 1 from the text. However, we were required to required to submit figures separately it was attached to the submission separately as per the PlosOne guidelines. We have added a statement to clarify the symptoms that we are assessing, a more detailed list of the symptoms is available in the measures section of the methods. The new sentence reads:

*“Figure 2 shows the 4-class base model’s probability of symptom endorsement across the core nine symptoms of depression in the DSM-5.”*

**4. In the attachments, figure 1 is missing explanations on the different colors in the figure.**

**Response:** As addressed in comments from reviewer 1, we completely agree on the issue of the figure legend, we have added this to the new version.

**5. Line 208: Class 3 (“moderate”) overall presented with 208 probabilities lower than Class 1 or 3 but higher than Class 4. I think it is a typing error where class 2 and 3 have been mixed up.**

**Response:** Thank you for noticing this error. We have made the necessary changes; it now reads as follows:

*“Class 3 (“moderate”) overall presented with probabilities lower than Class 1 or 2 but higher than Class 4.”*

**6. Line 238: Figure 2 is missing.**

**Response:** Thank you for your comment. We added the figure separately to the submission, as per the guidelines, instead of in-text. Our apologies that it was unavailable for you to view.
